# Anomalous Origin of Left Pulmonary Artery From the Descending Aorta Diagnosed in an Athletic Adult

**DOI:** 10.1016/j.jaccas.2025.103407

**Published:** 2025-04-02

**Authors:** Mariama Touray, Guillaume Fahrni, Tobias Rutz, Judith Bouchardy, Patrick Yerly, Aisha Touray, Magalie Ladouceur

**Affiliations:** a Cardiothoracic and Vascular Division, Lausanne University Hospital, Lausanne, Switzerland; b Department of Diagnostic and Interventional Radiology, University Hospital of Lausanne, Switzerland; c Cardiology Unit, University Hospitals of Geneva, Geneva, Switzerland

**Keywords:** anomalous origin of the left pulmonary artery (AOLPA), hemitruncus arteriosus, segmental pulmonary hypertension

## Abstract

**Background:**

Anomalous origin of the left pulmonary artery from the aorta, also named hemitruncus arteriosus, is a rare congenital heart disease associated with high mortality. Patients are usually operated on in the first months of life to avoid irreversible damage caused by pulmonary arterial hypertension.

**Case Summary:**

The authors present a challenging case of an athletic male patient with an anomalous left pulmonary artery originating from the descending aorta that was diagnosed when he was aged 27 years, with severe segmental pulmonary arterial hypertension in the left lung. Following multidisciplinary team meetings, conservative management was chosen.

**Discussion:**

Multimodality imaging plays a key role in both diagnosing and managing this birth defect and its potential complications.

**Take-Home Message:**

Management of this extremely rare congenital anomaly in the adult is yet to be fully understood and requires a multidisciplinary team in a tertiary center.

## Visual Summary

**Figure:**
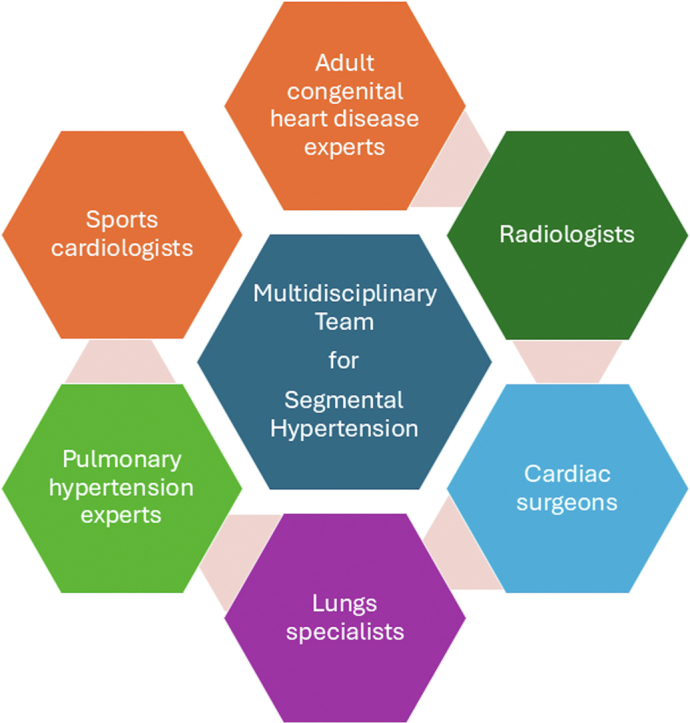

**Visual Summary:**
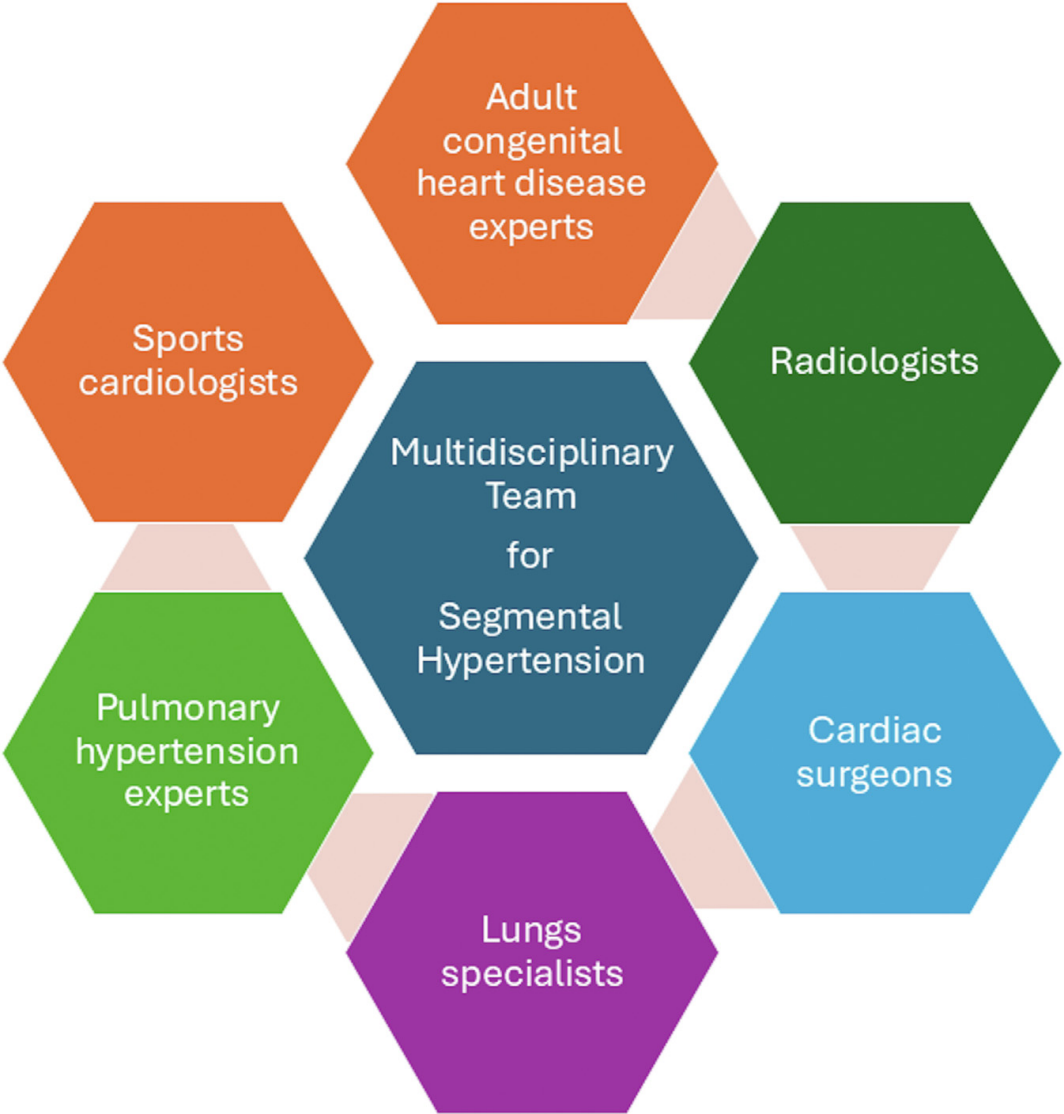
Anomalous Origin of the Left Pulmonary Artery in the Adult, Also Known as Hemitruncus Arteriosus

Anomalous origin of the left pulmonary artery (AOLPA) from the aorta, also named hemitruncus arteriosus (HA), is a rare congenital anomaly that constitutes 0.12% of congenital heart diseases (CHDs).^1^ In this CHD, the left lung is supplied by the systemic circulation, and in the long term, this leads to irreversible pulmonary vascular disease and pulmonary arterial hypertension (PAH).^2^ This disease can be isolated or associated with patent ductus arteriosus or tetralogy of Fallot.^2^ Most patients receive a diagnosis early in childhood, with a presentation of failure to thrive and heart failure symptoms. Without repair, mortality is high, reaching 70%, and only a few untreated patients survive into adulthood.^3^ Surgical repair is usually performed during the first 6 months of life to prevent PAH and improve survival.^4^ In the following case, we present an uncommon case of AOLPA that was diagnosed in an adult male athlete with segmental PAH of the left lung.

**Take-Home Messages**

• AOLPA, also known as hemitruncus, diagnosed in adulthood is associated with segmental PAH. The management of segmental PAH is challenging and requires a multidisciplinary approach at a tertiary center.
• Sports prescription in patients with PAH should be individualized according to clinical and hemodynamic parameters.

## History of Presentation

**Figure 1:**
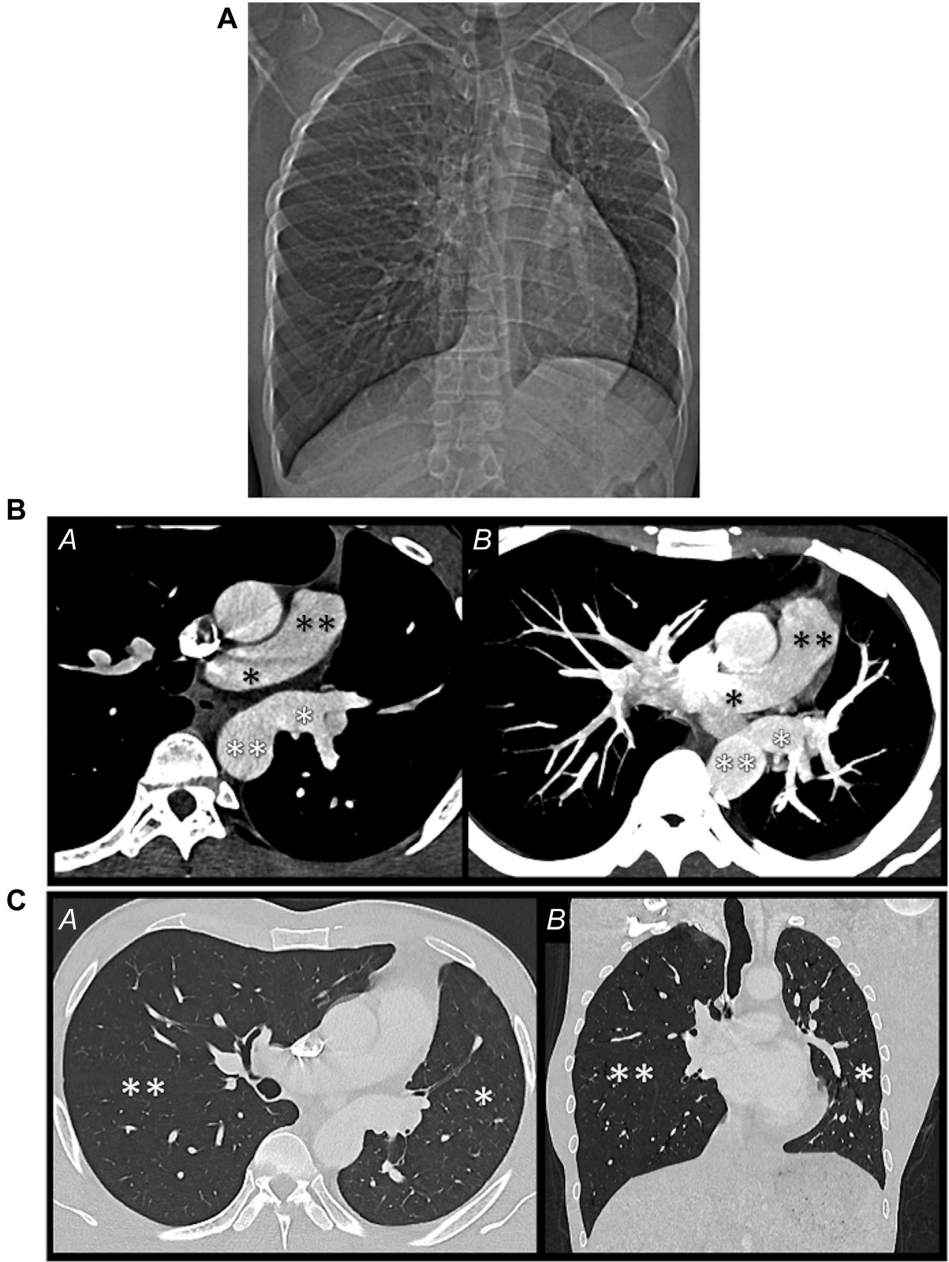
Computed Tomography Angiography (A) Scout view showing hypoplasia of the left lung and hyperinflation of the right lung. (B) Axial computed tomography angiography in the mediastinal window, from *(A)* top to *(B)* bottom, showing the right pulmonary artery (single black asterisk) and the pulmonary trunk (double black asterisk) and the left pulmonary artery (single white asterisk) from the descending thoracic aorta (double white asterisk). (C) *(A)* Axial and *(B)* coronal computed tomography angiography in the lung window showing hypoplasia of the left lung (single asterisk) and hyperinflation of the right lung (double asterisk).

A 27-year-old man, who was a former professional basketball player, was referred to the adult CHD (ACHD) unit for further investigation and management of an AOLPA from the descending aorta that was diagnosed on the basis of thoracic computed tomography angiography (CTA) (Figures 1A to C). The presenting sign that led to the consultation was chronic mild hemoptysis during exercise, which the patient engaged in on a daily basis. He reported no dyspnea and was classified as NYHA functional class I. Physical examination was unremarkable, with normal cardiac auscultation without cyanosis or hypoxemia.

## Past Medical History

The patient had no other medical history.

## Differential Diagnosis

The initial differential diagnosis of chronic hemoptysis included pulmonary parenchymal disorders, tracheobronchial anomalies, vascular disorders such as arteriovenous malformation, and infectious diseases (eg, tuberculosis).

## Investigations

The thoracic CTA at diagnosis revealed an AOLPA originating from the descending thoracic aorta and associated with global hypotrophy of the left lung with patchy ground-glass opacities, suggesting alveolar bleeding (Figures 1A to 1C).

**Figure 2:**
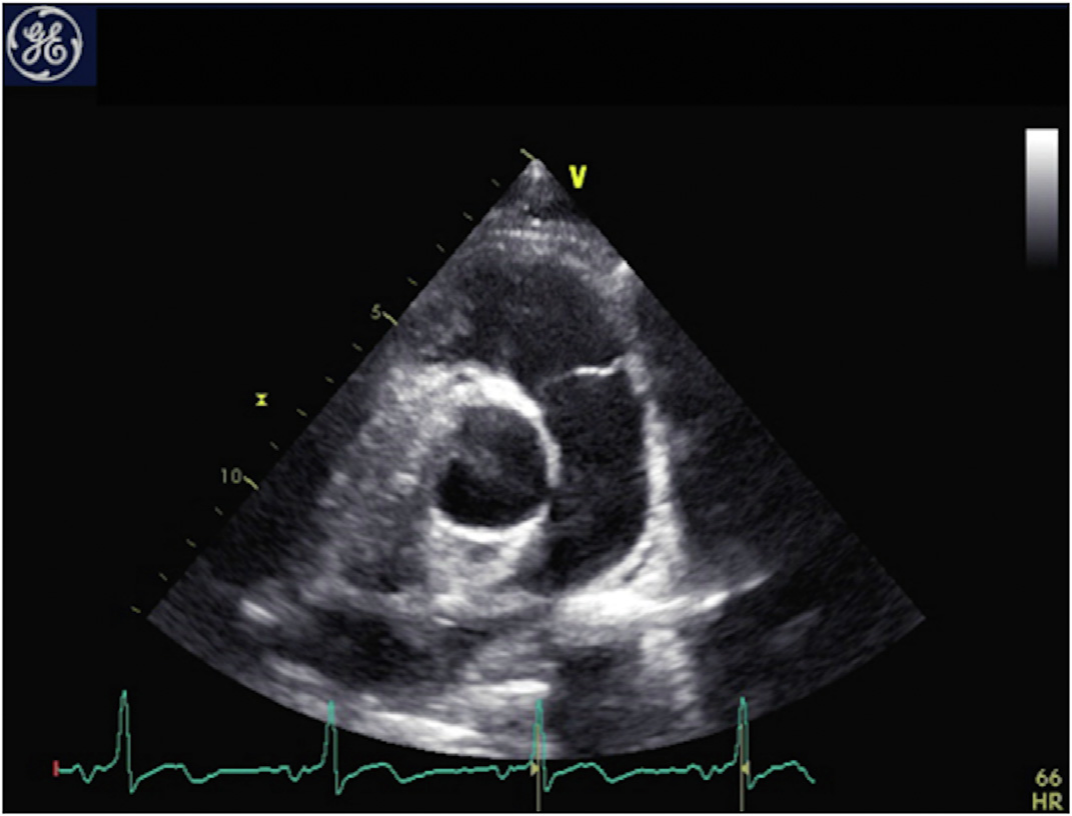
Echocardiography Parasternal short-axis view showing the absence of the left pulmonary artery.

The electrocardiogram showed normal sinus rhythm with early repolarization in the precordium from leads V_2_ to V_4_. His N-terminal pro–B-type natriuretic peptide level was normal, at 90 pg/mL. The transthoracic echocardiogram was remarkable for the absence of the left pulmonary artery (LPA) from the main pulmonary trunk, as depicted in Figure 2. No further associated congenital heart lesion was detected, and biventricular function was normal. There were no indirect signs of PAH in the right pulmonary artery (RPA).

Cardiac magnetic resonance (CMR) showed dilatation of both ventricles compatible with his high level of physical activity. Biventricular function was normal. The LPA rose from the descending aorta with a flow of 44 mL/beat or 3.0 L/min (RPA, 110 mL, 7.6 L/min). The pulmonary-to-systemic flow ratio (Q_p_/Q_s_ ratio) was calculated at 1.3, with Q_p_ estimated by pulmonary venous return and Q_s_ estimated by systemic venous return. The flow ratio between the RPA and the LPA was 71:39%.

Segmental PAH of the left lung was confirmed on cardiac catheterization, with a mean pulmonary artery pressure in the LPA of 82 mm Hg, as illustrated in Table 1. Pressures were normal in the RPA. Flow, as determined by CMR for each pulmonary artery, and respective pressure, as determined by cardiac catheterization, were used to calculate segmental pulmonary vascular resistance. LPA vascular resistance was estimated at 25.6 WU.

**Table 1:** Cardiac Catheterization: Left and Right Lung Separately

Heart rate, beats/min	60
Cardiac rhythm	Sinus rhythm
Pressure (s/d-m), mm Hg	
Ascending aorta	108/61-84
Descending aorta	108/61-81
PCWP	8/7-5
Right pulmonary artery	24/9-15
Left pulmonary artery	117/59-82
Right atrium	3/1-0
Cardiac flow in the right lung, L/min	8.1
Indexed cardiac flow in the right lung, L/min/m^2^	3.69
PVR right lung, WU	1.2
PVR left lung, WU^a^	25.6

Values are as stated.

PCWP = pulmonary capillary wedge pressure; PVR = pulmonary vascular resistance; s/d-m = systolic/diastolic-mean.

a To calculate the PVR in the left lung, we used the measured resting flow on cardiac magnetic resonance. Using a PCWP of 5 mm Hg—assumed to be the same in both lungs because both drain into the left atrium—the resistance was calculated at 25.6 WU. Direct measurement of PCWP in the left lung was avoided because of the high risk of hemoptysis when inflating the balloon.

Ergospirometry showed an obstructive syndrome with normal exercise capacity. The patient’s aerobic capacity was slightly reduced, with a peak oxygen uptake at 28.3 mL/kg/min, or 68% from the predicted value. Details of the respiratory function test results are illustrated in Table 2.

**Table 2:** Cardiopulmonary Exercise Testing

Spirometry	Predicted/Obtained Value	% of Predicted
VC, L	6.40/4.95	77
FEV_1_, L	5.04/3.21	64
FEV_1_/FVC, %	100/65	65
FEV_1_/VC, %	100/65	65
PEF, L/s	10.86/6.65	61

**Ergospirometry**	**Predicted/Obtained Value at Peak**	

RER	1.21/1.16	96
Watts	243/209	86
Vo_2_max, mL/min/kg	41.3/28.3	68
Maximum heart rate, beats/min	192/155	81
Systolic blood pressure, mm Hg	207/150	72

Values are as stated and % of predicted.

FEV_1_ = forced expiratory volume in 1 second; FVC = forced vital capacity; PEF = peak expiratory flow; RER = respiratory exchange ratio; VC = vital capacity; V**o**_2_max = maximal oxygen uptake.

## Management

Following multidisciplinary team meetings, including ACHD specialists, PAH experts, and sports cardiologists, a conservative management approach was chosen, with annual follow-up. Surgical repair, aimed at restoring normal anatomy by reimplanting the LPA into the main pulmonary trunk, was not pursued because of irreversible PAH in the left lung. Currently asymptomatic, the patient does not receive treatment for PAH. In the event of recurrent hemoptysis, percutaneous coil embolization of the responsible artery would be considered. Because hemoptysis was triggered by sustained exercise, high-intensity activities were discouraged, but mild to moderate activities were prescribed.

## Follow-Up

The patient did not experience any new bleeding over the following 6 months.

## Discussion

We present a challenging case of an athletic 27-year-old man with segmental PAH of the left lung secondary to an AOLPA from the aorta. We highlight the key role of multimodality imaging—echocardiography, CTA, CMR, and invasive angiography—in diagnosing and managing this congenital defect. A single imaging modality is often insufficient, particularly when faced with a broad differential diagnosis, such as the LPA arising from the right (pulmonary artery sling) on echocardiography. CMR and cardiac catheterization were crucial in evaluating the pulmonary pressures and resistance of the left lung. CTA not only plays a fundamental role in diagnosis but also guides treatment options for complications such as hemoptysis, which is frequently encountered. In most patients, the diagnosis is made during early childhood, and prompt surgical treatment is recommended to improve survival and prevent irreversible damage caused by prolonged PAH.^4^ Nathan et al^5^ showed excellent surgical outcomes in patients operated on within the first 6 months of life, with 93% survival at 2 years.

The present patient is inoperable and is eligible only for a future pneumonectomy in case of recurrent hemoptysis even after embolization. Corno et al^6^ studied the physiopathology of this congenital anomaly in experimental pig models. His team established that exposure of the lung to chronic systemic pressure and oxygenation leads to irreversible vascular lesions typical of pulmonary vascular obstructive disease.

In the present case, invasive catheterization measures confirmed the presence of PAH isolated to the left lung. Dimopoulos et al^3^ defined this entity as segmental PAH and currently included it under the umbrella of World Heart Organization group 1. Treatment for segmental PAH remains challenging because of the rarity and lack of evidence.^3^

Vasoreactivity tests were not performed in the present case. These tests are typically done to identify patients who could benefit from high-dose calcium-channel blocker therapy. Currently, high-dose calcium-channel blocker therapy is approved for patients with idiopathic PAH, drug-induced PAH, or hereditary PAH.^7^ In PAH in the context of CHD with systemic-to-pulmonary shunting, vasoreactivity testing can be performed to evaluate the possibility of defect closure.^7^ The patient has a strict contraindication to defect repair given the markedly elevated pulmonary vascular resistance in the LPA, combined with a nonsignificant systemic-to-pulmonary shunt.^7^

Medical treatment is considered in symptomatic patients with segmental PAH who are not eligible for surgery or percutaneous procedures;^8^ however, debate on this matter persists.^3^^,^^7^ The PAH therapies studied comprise bosentan, (a dual endothelin receptor antagonist), prostanoids, and sildenafil (a phosphodiesterase-5 inhibitor).^3^^,^^6^^,^^7^

Schuuring et al^8^ showed a significant improvement in functional class and exercise capacity in patients with segmental PAH who received bosentan treatment. These patients were all symptomatic at baseline, with NYHA functional class II or III.^8^ Lim et al^9^ also demonstrated a symptomatic improvement in patients with segmental PAH.

Until recently, little was known about exercise training in patients with PAH, and sports participation was often discouraged. However, more current studies have demonstrated the beneficial effects of exercise training on exercise capacity, quality of life, muscular function, and possibly pulmonary hemodynamics.^10^ We prescribed sports participation according to the algorithm provided by Budts et al^10^ that mainly takes into account 5 parameters: assessment of ventricular function and anatomy, PAH, aorta, arrhythmia and arterial oxygen saturation. Because of the presence of PAH in the present patient, we discouraged high-intensity and competitive sports. However, in the absence of ventricular dysfunction, we prescribed mild- to moderate-intensity activities involving skill, power, and/or mixed sports.^10^

## Conclusions

Our case highlights the challenges concerning the treatment options in AOLPA from the aorta diagnosed in a young athletic adult. This entity requires a multidisciplinary team discussion in a tertiary center with ACHD and PAH experts.

## Funding Support and Author Disclosures

The authors have reported that they have no relationships relevant to the contents of this paper to disclose.
